# Integration of Bulk and Single-Cell RNA Sequencing Analyses in Biomedicine

**DOI:** 10.3390/ijms27073334

**Published:** 2026-04-07

**Authors:** Nikita Golushko, Anton Buzdin

**Affiliations:** 1 Scientific Center of Genetics and Life Sciences, Sirius University of Science and Technology, 354340 Sirius Federal Territory, Russia; golushko.ni@talantiuspeh.ru; 2 Institute for Personalized Oncology, Biomedical Science & Technology Park, Sechenov First Moscow State Medical University, 119991 Moscow, Russia

**Keywords:** bulk RNA sequencing (bulk RNAseq), single-cell RNA sequencing (scRNAseq), data integration, transcriptomic deconvolution, cell-type atlas, pseudobulk, batch effects, Bayesian modeling

## Abstract

Transcriptome profiling is a cornerstone of functional genomics, enabling the detailed characterization of gene expression in health and disease. Bulk RNA sequencing (bulk RNAseq) remains the most widely used approach in clinical and large-cohort studies due to its cost-effectiveness, robustness, and comprehensive transcriptome coverage. However, bulk RNAseq inherently averages gene expression signals across heterogeneous cell populations, thereby masking cellular diversity and obscuring rare cell types. In contrast, single-cell RNA sequencing (scRNAseq) enables a high-resolution analysis of cellular heterogeneity, allowing the identification of distinct cell types, transitional states, and developmental trajectories. Nevertheless, scRNAseq is associated with higher cost, limited scalability, increased technical noise, sparse expression matrices, and protocol-dependent biases introduced during tissue dissociation or nuclear isolation. In this review, we summarize the conceptual and methodological foundations of integrating bulk RNAseq and scRNAseq data, emphasizing their complementary strengths and limitations. We discuss how scRNAseq-derived cell-type atlases can serve as reference matrices for computational reconstruction (deconvolution) of bulk RNAseq profiles and examine key sources of technical and biological variability. Furthermore, we outline major integration strategies, including reference-based deconvolution, pseudobulk aggregation, and Bayesian joint modeling to provide an overview of widely used analytical tools and essential components of scRNAseq data processing workflows.

## 1. Introduction

Transcriptome profiling has become an indispensable tool in modern functional genomics, enabling the investigation of the molecular mechanisms underlying cellular processes in both physiological and pathological conditions. Traditional bulk RNA sequencing (bulk RNAseq) provides high transcriptome coverage and strong statistical power for gene expression analysis. However, bulk RNAseq averages signals across heterogeneous cell populations, thereby masking intercellular variability in gene expression [1,2]. The emergence of single-cell RNA sequencing (scRNAseq) technologies has revolutionized the field, opening unprecedented opportunities for both quantitative and qualitative investigation of cellular heterogeneity within tissues, as well as for unbiased identification and analysis of diverse cell populations, including rare subsets [3].

At the same time, these advances in scRNAseq are accompanied by substantial technical limitations, including high cost, technical noise, experimental complexity, and incomplete transcriptome coverage at the single-cell level [4]. Therefore, the integration of bulk RNAseq and scRNAseq holds strong potential to overcome the limitations inherent to each approach when used independently. In particular, scRNAseq data can serve as reference cell-type atlases for functional reconstruction (deconvolution) of bulk RNAseq profiles, enabling an estimation of tissue cellular composition and the detection of its alterations across different conditions, including pathological states [5,6]. Such integrative strategies have already been widely applied in oncology [7,8], immunology [9,10], neurobiology [11], and other biomedical fields where understanding cellular heterogeneity is essential for deciphering disease mechanisms and developing modern therapeutic approaches.

Despite the rapid development of scRNAseq technologies and the accumulation of increasingly large datasets across diverse tissues and organs [12], the vast majority of tissue samples are still characterized using bulk RNAseq. This is due to both economic factors, as the cost of scRNAseq remains substantially higher and technical limitations most frequently requiring the use of fresh or cryopreserved samples, which complicates retrospective studies and routine clinical practice [13,14]. As a result, a critical gap has emerged between the detailed understanding of cellular heterogeneity obtained from scRNAseq experiments and the large-scale, clinically relevant data available in the form of bulk RNAseq profiles. The integration of these two data types through so-called computational deconvolution makes it possible to address several key challenges simultaneously.

First, it enables a quantitative estimation of tissue cellular composition in large clinical cohorts, linking shifts in the proportions of specific cell populations to clinical outcomes, therapeutic response and prognostic factors [5,6]. Second, deconvolution allows the identification of cell-type-specific transcriptional programs and signaling pathways active in particular pathological conditions, which is essential for understanding disease mechanisms [15]. Third, integrative analysis creates opportunities for the development of novel biomarkers and therapeutic targets based on cell-type-specific gene expression patterns [16].

This integration is particularly relevant in immuno-oncology, where the composition and functional state of cells within the tumor microenvironment largely determine the efficacy of immunotherapy [17]. Deconvolution of bulk RNAseq data enables the estimation of tumor infiltration by different immune cell types, identification of immunosuppressive populations, and prediction of response to immune checkpoint inhibitors. Similar approaches are applied in neurobiology to investigate the cellular composition of different brain regions in neurodegenerative diseases [11]. In Alzheimer’s disease, for example, alterations in the bulk transcriptome have been shown to reflect both changes in transcriptional regulation and shifts in cellular composition, including reduced neuronal abundance and increased proportions of microglia and astrocytes. These alterations substantially influence differential expression patterns of genes specific to distinct cell types [18].

Deconvolution methods for bulk RNAseq based on single-cell reference datasets (e.g., CIBERSORTx [19]) enable the simultaneous estimation of changes in cellular composition by calculating relative proportions of dozens of cell types and reconstruction of cell-type-specific expression profiles. Technological differences between scRNAseq and bulk RNAseq, including library preparation protocols, RNA capture efficiency, sequencing depth, and pronounced batch effects, as well as the intrinsic technical noise and transcript dropout events characteristic of single-cell datasets, may introduce substantial challenges in data analysis [20]. Moreover, the very process of estimating cell-type proportions may introduce additional biases and complicate the statistical interpretation of the results [21]. Therefore, in the following sections, we examine the current approaches to transcriptome profiling, their experimental principles, computational analysis algorithms, and the major strengths and limitations that ultimately determine the appropriate selection and optimization of data integration strategies.

Several recent publications have addressed bulk RNAseq and scRNAseq from broader computational, technological, or disease-oriented perspectives. For example, a recent review by Tzec-Interián et al. [22] provided a systematic comparison of computational workflows for bulk and single-cell transcriptomics, whereas Li and Wang [23] discussed the evolution from bulk to single-cell and spatial RNA sequencing in precision oncology. In addition, recent original studies have illustrated the practical value of integrating bulk RNAseq and scRNAseq for disease-specific biomarker discovery and prognostic modeling. Specifically, Mou [24] and Harries applied scRNAseq to assign cell-type identities to genes consistently dysregulated in bulk prostate cancer datasets, subsequently using this cellular annotation to inform the construction of an epithelial marker gene signature for biochemical recurrence prediction via machine learning; CIBERSORT was employed as a downstream tool for immune cell abundance estimation rather than as a method for systematically integrating bulk and single-cell data. However, none of these publications provides a systematic methodological comparison of integration strategies as a distinct analytical discipline: algorithmic assumptions, quantitative benchmarking data, cross-platform confounders, and structured guidance for context-dependent method selection are not addressed in a unified framework. In the present review, we fill this gap by focusing on the principal strategies for integrating bulk RNAseq with scRNAseq (including reference-based deconvolution, pseudobulk aggregation, and Bayesian joint modeling) providing a structured comparison of their algorithmic assumptions, quantitative performance, computational requirements, and practical applicability, together with explicit guidance for context-dependent method selection that is not available in the current literature.

## 2. Methods of Transcriptome Analysis

### 2.1. Bulk RNA Sequencing: Tissue-Level Profiling

Bulk RNAseq is a high-throughput method that enables a quantitative assessment of gene expression in a tissue sample or a population of cells [1,25]. The approach is based on the extraction of total RNA from a biological specimen, followed by reverse transcription into complementary DNA (cDNA) and high-throughput parallel sequencing of the resulting fragments [26]. Gene expression levels are estimated based on the number of sequencing reads mapped to individual genes. For a quantitative comparison of expression levels across genes, normalization procedures are required to account for transcript length, sequencing depth, and other technical factors [2,27]. Bulk RNAseq protocols can be implemented using various technological platforms, including Illumina, MGI, GeneMind, Oxford Nanopore, and PacBio, each characterized by distinct read lengths, accuracy profiles, and throughput capacities [28,29,30,31].

A standard bulk RNAseq protocol includes RNA or cDNA fragmentation, adapter ligation, library amplification, and sequencing, followed by bioinformatic processing steps such as quality control, alignment to a reference genome, and the quantitative estimation of gene expression levels [2,32,33]. The principal advantages of bulk RNAseq include high transcriptome coverage, relatively affordable cost per sample, strong reproducibility across technical replicates, and the broad dynamic range of transcript detection [1,34,35,36,37]. These properties make bulk RNAseq well-suited for large-scale clinical and cohort studies, as discussed further below. Over the past 15 years, large-scale bulk RNAseq datasets have been accumulated in publicly available repositories such as The Cancer Genome Atlas (TCGA), Genotype–Tissue Expression (GTEx), and Gene Expression Omnibus (GEO), which now represent primary targets for retrospective integrative analyses [38,39,40].

A key limitation of bulk RNAseq is the averaging of transcriptomic signals across all cells within a sample, resulting in a loss of information about cellular heterogeneity. This is particularly critical in complex tissues such as tumors, where multiple cell types with markedly distinct transcriptomic profiles coexist [41]. As a consequence, rare cell populations may remain undetected and their contributions to biological processes may be diluted or underestimated [6]. For example, the tumor microenvironment contains fibroblasts, endothelial cells, various immune cell types (T cells, macrophages, and dendritic cells), as well as malignant cells, all of which may exist in different functional states [42]. In addition, tumors may contain a fraction of adjacent normal tissue cells [43]. Bulk RNAseq, therefore, provides an averaged expression profile of all these cell types, complicating the interpretation of the results and limiting insights into molecular mechanisms specific to individual cellular populations [44]. Moreover, dynamic processes such as cellular differentiation [45], immune activation [46], and epithelial–mesenchymal transition [47] are characterized by heterogeneous cellular states that are entirely masked in bulk-level analyses.

This limitation is particularly pronounced in the context of tumor heterogeneity, where distinct subpopulations of cancer cells with diverse molecular characteristics may determine therapeutic resistance and metastatic potential [48,49]. Bulk RNAseq data do not allow a direct assessment of intercellular interactions and communication between different cell types, as this approach lacks the cellular resolution required for the analysis of ligand–receptor interactions [50]. The inability to directly analyze cellular composition and intercellular communication has, in turn, driven the development of computational deconvolution methods. These approaches are specifically designed to compensate for the lack of cellular resolution and to reconstruct the relative abundance of different cell populations in heterogeneous samples [51,52].

**Figure 1:**
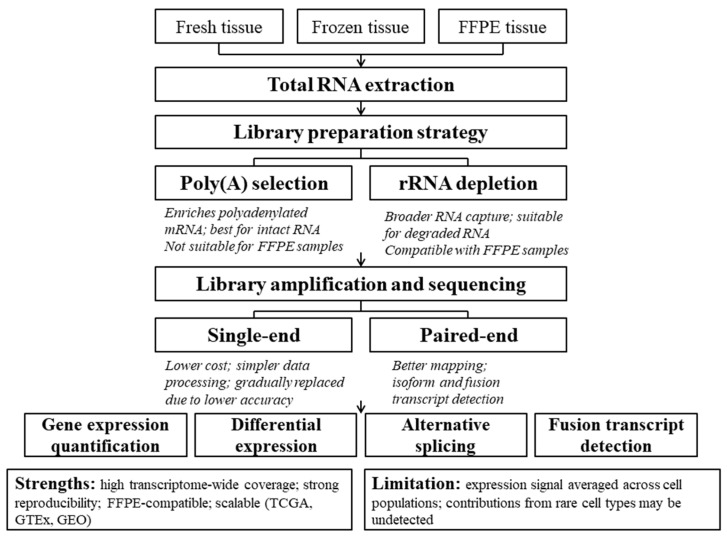
Overview of the bulk RNAseq workflow and major analytical outputs. Bulk RNAseq starts from total RNA extracted from fresh, frozen, or FFPE tissue, followed by library preparation using either poly(A) selection or rRNA depletion, library amplification, and single-end or paired-end sequencing. The resulting data support gene expression quantification, differential expression analysis, alternative splicing analysis, and fusion transcript detection. The scheme also summarizes major strengths of bulk RNAseq, including broad transcriptome coverage and scalability, as well as its principal limitation, namely the loss of cellular resolution due to signal averaging across heterogeneous cell populations.

For example, algorithms such as CIBERSORT [51] and EPIC [53] use reference gene expression profiles derived from purified cell populations of a single type to quantitatively estimate the immune and tumor composition of tissues based on aggregated transcriptomic data. These methods rely on predefined cell-type-specific reference profiles generated either from purified cell populations, as noted above, or from scRNAseq data. This imposes important limitations, as it requires prior knowledge of transcriptomes from individual cell types. It also makes the resulting estimates of cellular composition vulnerable to technological discrepancies between bulk RNAseq and scRNAseq workflows, including gene-specific biases arising from differences in target RNA capture protocols and library preparation procedures [54]. An overview of the bulk RNAseq workflow is shown in Figure 1.

### 2.2. Single-Cell RNA Sequencing: Diversity of Cell Types

The emergence of scRNAseq marked a fundamentally new stage in transcriptomics, enabling the transition from averaged population-level measurements to gene expression analysis at single-cell resolution. This conceptual shift was first demonstrated in [3], where complete transcriptome profiles of individual cells were obtained, revealing gene expression levels, alternative splicing features, and cellular heterogeneity that were not resolvable using previously applied bulk sequencing approaches. Conceptually, scRNAseq is based on the isolation of individual cells or their nuclei, capture of their RNA, and deep sequencing, in which each RNA molecule is labeled with unique molecular identifiers (UMIs) and cell-specific sequences known as barcodes. This enables the accurate quantification of gene expression while simultaneously preserving the cellular origin of each transcript [55]. The evolution of scRNAseq technologies reflects the pursuit of an optimal balance between per-cell sequencing depth and experimental scalability.

Plate-based formats, in which individual libraries are prepared for each cell in separate wells, are characterized by relatively low throughput and high cost, typically limiting analysis to only several hundred cells [56,57]. A major technological advance toward increased scalability was the introduction of droplet-based microfluidics, exemplified by the Drop-seq method [58].

More recently, probe-based single-cell transcriptomic approaches have further expanded the range of feasible experimental designs. For example, 10x Genomics Fixed RNA Profiling/Flex workflows enable targeted transcript detection in fixed samples, thereby improving compatibility with complex study designs and facilitating larger-scale applications in settings where conventional whole-transcriptome scRNAseq may be technically challenging. At the same time, these methods are restricted to predefined probe panels covering protein-coding genes of supported species and therefore do not capture non-coding transcripts or enable unbiased de novo transcript discovery, which should be considered when selecting an appropriate strategy for integrative analyses [59].

Discoveries enabled by scRNAseq have fundamentally transformed our current understanding of cellular biology. For example, in immunology, the application of scRNAseq refined the taxonomy of human blood dendritic cells and monocytes [60]. In addition to previously recognized subtypes, novel populations were identified, including a rare population of AXL^+^SIGLEC6^+^ blood dendritic cells positioned between plasmacytoid dendritic cells (pDCs) and CD1C^+^ conventional dendritic cells (cDCs). In the context of antitumor immunity, scRNAseq revealed that exhausted CD8^+^ T cells comprise two major populations: progenitor-like cells that retain longevity, proliferative capacity, and sensitivity to PD-1 blockade, terminally exhausted cells that are more cytotoxic but short-lived and exhibit minimal expansion during PD-1-targeted immunotherapy [61].

At the same time, intrinsic technical characteristics of scRNAseq impose fundamental limitations on data interpretation. Because the initial amount of mRNA within a single cell is extremely low, library preparation in scRNAseq requires amplification by more than a million-fold, introducing substantial nonlinear distortions. These include preferential amplification of certain transcripts and significant alteration in their relative abundances, thereby increasing technical variability in the data [62].

The low initial amount of mRNA increases the probability that individual transcripts will be missed during reverse transcription and therefore not detected during sequencing, resulting in so-called dropout events, where a gene expressed at moderate or high levels in one cell may appear completely absent in another [63]. Statistical modeling of UMI counts in controlled droplet-based experiments has shown that the proportion of zero values for each gene is well-described by a negative binomial distribution with a shared dispersion parameter across genes. The observed excess of zeros in biological samples is not solely attributable to technical limitations of the method but is largely explained by biological variability and cellular heterogeneity [64].

Biological artifacts introduced during sample preparation add an additional layer of complexity to data interpretation. Dissociation of solid tissues into individual cells induces a pronounced transcriptional stress response characterized by substantial activation of immediate early response genes (e.g., *Fos*, *Jun*) and heat shock genes (*Hspa1a*, *Hspa1b*, *Hspb1*), as demonstrated, for example, in studies of muscle stem cell models [65]. Moreover, the magnitude of this response varies considerably across cell and tissue types.

**Figure 2:**
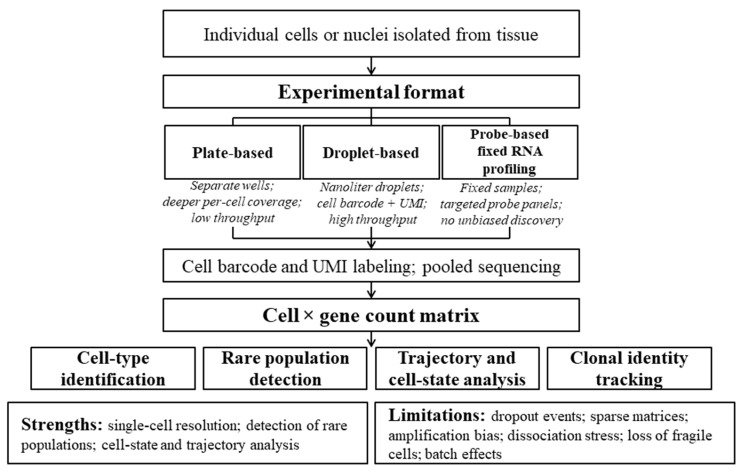
Overview of the scRNAseq workflow, experimental formats, and major analytical applications. scRNAseq begins with the isolation of individual cells or nuclei, followed by platform-specific library preparation using plate-based, droplet-based, or probe-based fixed-RNA workflows; cell barcode and UMI labeling; and pooled sequencing to generate a cell-by-gene count matrix. The resulting data enable cell-type identification, rare population detection, trajectory and cell-state analyses, and clonal identity tracking. The scheme also summarizes the main strengths of scRNAseq, including single-cell resolution and its major limitations, such as dropout events, sparse matrices, amplification bias, dissociation-induced stress, loss of fragile cell types, and batch effects.

An alternative strategy is single-nucleus RNA sequencing (snRNAseq), which enables the effective preservation of “fragile” cell types that are sensitive to enzymatic dissociation. However, this approach is characterized by a shift in transcriptomic reads toward nuclear RNA and intronic sequences, leading to the relative depletion of cytoplasmic transcripts such as mRNA [66,67]. An overview of the scRNAseq workflow is shown in Figure 2.

### 2.3. Strengths and Limitations of Each Approach

A comparative analysis of bulk RNAseq and scRNAseq highlights their complementary roles in modern transcriptomics and the key differences between these approaches are summarized in Table 1. In standard experiments, bulk RNAseq libraries are typically sequenced to a depth range of 10–30 million reads per sample, providing robust quantitative gene expression data across the transcriptome [33]. According to our data, high-quality analysis of protein-coding gene expression in human tissues can be achieved with more than 3.5 million reads uniquely mapped to such genes [37]. In most cases, a sequencing depth range of 20–30 million reads is fully sufficient for this purpose. Notably, the threshold for adequate sequencing depth in analyses of cell line-derived samples is generally even lower.

According to the results of the SEQC (MAQC-III) project [72], bulk RNAseq demonstrates high inter-laboratory reproducibility when standardized protocols are applied: correlations of relative gene expression levels across sites and platforms typically exceed 0.9 and after filtering by fold change and expression level, the overlap of differentially expressed gene lists reaches approximately 95%. The SEQC findings also emphasize the important role of technical replicates in improving analytical reliability, although the optimal number of such replicates was not specified [72]. In our own experiments, analysis of identical RNA samples extracted from FFPE tumor and pathological tissues, sequenced independently in different laboratories on different continents (with new library preparation performed in each case) but using the same protocol, yielded correlations between gene expression profiles in the range of 0.95–0.96 [37,79]. On the other hand, the unprecedented ability to profile tens of thousands of individual cells in parallel within a single experiment has enabled the construction of comprehensive cell atlases of tissues and organs, as well as the discovery of previously unrecognized cell types and functional states that cannot be identified using bulk tissue-level approaches.

The specific characteristics of each approach determine their optimal areas of application. For example, when analyzing heterogeneous tissues, bulk RNAseq cannot distinguish whether observed changes in gene expression are driven by shifts in cell-type proportions, by transcriptional alterations within specific cell populations, or by a combination of both factors [80]. At the same time, different scRNAseq protocols exhibit substantially varying efficiencies of intracellular RNA capture, resulting in marked differences in library complexity, representation of lowly expressed transcripts, and the frequency of dropout events (the stochastic zero-expression phenomenon discussed above).

Moreover, different methods exhibit pronounced technical variability, with a substantial proportion of gene expression variance attributable to the specific protocol used. This complicates data integration and necessitates more rigorous strategies for harmonization of transcriptomic profiles [73]. Modern scRNAseq data analysis requires considerable computational resources and familiarity with a rapidly expanding methodological landscape. As of 2023, more than 1400 bioinformatic tools have been developed for scRNAseq data analysis [81]. At the same time, many key analytical parameters and processing steps remain insufficiently standardized and several routinely addressed technical tasks lack independent benchmarking assessments and unified best-practice workflows. Furthermore, the requirement for fresh or appropriately cryopreserved tissue samples further limits the applicability of scRNAseq.

In contrast, bulk RNAseq results remain technically reproducible, reliably interpretable and clinically meaningful even when applied to degraded RNA extracted from FFPE tissue samples stored at room temperature for several years [82,83]. Thus, neither method represents a universal solution. Their integration through analytical approaches enables the strengths of each to be leveraged in order to achieve a more comprehensive understanding of the transcriptomic state of the biological system under investigation. In the following section, we discuss the theoretical foundations and practical strategies for integrating bulk and single-cell transcriptomic profiling data, including the construction of cell-type-specific reference expression matrices, deconvolution of bulk RNAseq profiles, and pseudobulk gene expression analysis. We also review computational algorithms used for data integration and their applications in addressing biomedical research questions.

## 3. Data Integration

Joint profiling of biological samples using bulk RNAseq and scRNAseq exploits the complementary strengths of both technologies, as outlined in Section 2.1, Section 2.2 and Section 2.3, to achieve more accurate and comprehensive transcriptomic characterization.

### 3.1. Importance of Integrating Bulk and Single-Cell RNA Sequencing Data

As discussed in Section 2.1, Section 2.2 and Section 2.3, the limitations of bulk and single-cell RNA sequencing are largely complementary: bulk RNAseq lacks cellular resolution but offers cost-effectiveness and scalability, while scRNAseq resolves cellular heterogeneity but remains constrained by technical noise, dropout, dissociation artifacts, and limited applicability to large clinical cohorts [44,75,84]. These complementary properties provide a strong rationale for joint analysis, as the integration of both data types allows simultaneous compensation for the principal technical limitations of each approach [85].

Moreover, the integration of scRNAseq data with spatial transcriptomics datasets, conceptually similar to bulk RNAseq results in terms of aggregated signal, enables the transfer of single-cell level information onto spatially resolved transcriptomic data [86,87]. Spatial transcriptomics preserves tissue architecture and can be integrated with scRNAseq-derived reference atlases to assign cell types to spatial locations, estimate their local abundance, and, in some cases, reconstruct broader spatial gene expression patterns. Representative methods include Tangram, which aligns sc/snRNAseq and spatial transcriptomic data to generate transcriptome-wide spatial maps and support single-cell resolution reconstruction from lower-resolution platforms and Cell2location, which uses a Bayesian framework to infer fine-grained cell-type composition in spatial transcriptomic profiles [88,89].

Beyond spatial transcriptomics, an additional important direction of integrative analysis involves multimodal single-cell reference datasets that jointly profile transcription together with complementary molecular layers, such as cell-surface protein abundance or chromatin accessibility. For example, CITE-seq enables the parallel measurement of mRNA and surface epitopes in the same cells, improving phenotypic resolution beyond transcriptome data alone [90]. More recently, TEA-seq extended this concept by enabling the simultaneous measurement of transcripts, epitopes, and chromatin accessibility, thereby linking gene expression states to both phenotypic markers and putative regulatory programs within individual cells [91]. In parallel, computational frameworks such as GLUE have been developed to integrate heterogeneous single-cell multi-omics datasets across distinct feature spaces and to support regulatory inference from combined transcriptomic and epigenomic information [92].

**Figure 3:**
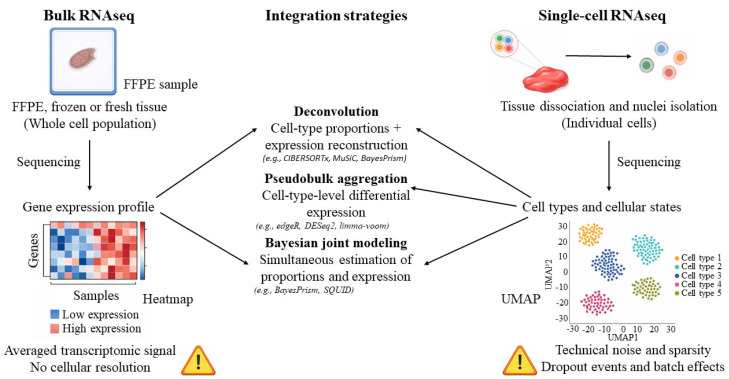
Conceptual overview of bulk and scRNAseq integration strategies. Bulk RNAseq provides an averaged tissue-level gene expression profile derived from FFPE, frozen or fresh tissue samples, without cellular resolution. In contrast, scRNAseq enables the characterization of individual cell types and cellular states following tissue dissociation or nuclei isolation, but is affected by technical noise, dropout events, and batch effects. Integration strategies include: reference-based deconvolution (e.g., CIBERSORTx, MuSiC, BayesPrism), enabling the estimation of cell-type proportions and reconstruction of cell-type-specific expression profiles from the bulk expression matrix using a scRNAseq-derived reference; pseudobulk aggregation (e.g., edgeR, DESeq2, limma-voom), where single-cell data are aggregated at the cell-type level to enable differential expression analysis using bulk RNAseq statistical frameworks; and Bayesian joint modeling (e.g., BayesPrism, SQUID), which uses scRNAseq data as a probabilistic prior and simultaneously estimates cell-type proportions and cell-type-specific expression profiles across platforms. UMAP (Uniform Manifold Approximation and Projection) represents a nonlinear dimensionality reduction method used for visualization of single-cell transcriptomic data; cell-type labels in the UMAP legend are shown schematically. The heatmap illustrates a gene expression matrix (genes vs. samples) derived from bulk RNAseq, where color intensity reflects relative expression levels. Arrows indicate the direction of data flow from each transcriptomic modality to the corresponding integration strategy.

Such integration helps compensate for technological limitations of spatial platforms and improves the accuracy of tissue cellular composition interpretation [93]. In addition, scRNAseq facilitates the identification of cell-type-specific gene expression drivers that may be difficult to detect in bulk RNAseq data. This substantially expands analytical opportunities across a wide range of applications, including the characterization of the tumor microenvironment, immune states, and tissue architecture in population-scale studies [94]. In the Section 3.2, we outline the principal strategies for integrating heterogeneous transcriptomic datasets. The complementary strengths and limitations of bulk RNAseq and scRNAseq, as well as the principal strategies for their integration, are summarized schematically in Figure 3.

### 3.2. Major Approaches to Integrating Single-Cell and Bulk RNA Sequencing Data: Deconvolution and the Pseudobulk Strategy

A fundamental component of integrating distinct classes of transcriptomic data is deconvolution, a procedure that decomposes aggregated tissue-level transcriptomic profiles into contributions from individual cell types using scRNAseq-derived reference datasets. Among widely used contemporary deconvolution methods are CIBERSORTx, MuSiC, Bisque, and Kassandra, which apply diverse statistical and machine learning-based approaches to accurately reconstruct cellular composition from bulk transcriptomic profiles.

CIBERSORTx applies support vector regression with platform-specific normalization and batch effect correction to minimize bias when comparing scRNAseq and bulk RNAseq data [6]. MuSiC employs weighted non-negative regression that accounts for inter-individual and intra-cell-type variability of marker genes, combined with a hierarchical procedure for identifying closely related cell types [95]. Bisque implements gene-specific linear transformations to correct for technical discrepancies between scRNAseq and bulk RNAseq expression measurements, substantially improving deconvolution accuracy in the presence of systematic bias [54]. Kassandra is based on a computational decision tree model trained on millions of artificially generated transcriptomes, enabling the model to capture both biological and technical variability and to provide relatively robust estimates of cell-type proportions across diverse tissue contexts [96].

In addition to regression-based, probabilistic, and tree-based frameworks, deep learning-based deconvolution has emerged as an additional direction in bulk–single-cell integration. These methods are typically trained on synthetic bulk transcriptomes generated from scRNAseq data to learn nonlinear relationships between bulk expression and cellular composition. Scaden is a representative example of this approach [97]. Across all these methods, accurate selection of marker genes and appropriate modeling of expression variability and inter-individual effects are central to improving deconvolution performance. The importance of deconvolution is particularly pronounced in structurally complex tissues characterized by high cellular heterogeneity, as demonstrated by scRNAseq data [94].

In addition to reference-based deconvolution, reference-free approaches have also been developed for cases where no suitable single-cell or purified-cell reference is available. These methods aim to infer cellular structure directly from bulk transcriptomic data by exploiting latent expression patterns, feature variability or cell-type-specific signals without predefined signatures. Representative examples include Linseed, which reconstructs mixture structure based on transcriptional simplex geometry and mutual linearity of marker genes [9] and TOAST, which iteratively improves reference-free cell composition estimation through cross-cell-type differential analysis and feature selection [98]. Although reference-free methods are especially useful for poorly characterized tissues, their performance is generally less stable and their biological interpretation more difficult than in reference-based frameworks.

An important practical challenge shared by deconvolution-based integration strategies is batch-effect confounding between bulk RNAseq datasets and scRNAseq-derived references [99]. Deconvolution performance may decline substantially in realistic cross-reference settings, where bulk data and single-cell references originate from different donors, studies, batches, or sequencing platforms, compared with idealized self-reference benchmarks based on the same dataset [100]. Such mismatch may affect not only transcriptional profiles but also the observed cell proportions in reference data, thereby introducing additional uncertainty into the estimated cellular composition of bulk samples [100]. Importantly, this discrepancy is not limited to batch effects in the narrow sense, but may also reflect broader cross-platform biases between bulk and single-cell measurements. In particular, differences in cell-type-specific mRNA content may systematically distort estimated cell fractions if not explicitly accounted for, because cell populations with higher or lower total mRNA abundance can be over- or underestimated during deconvolution [101].

In contrast, an important component of modern differential gene expression analysis strategies in scRNAseq datasets is the so-called pseudobulk approach, in which sequencing reads assigned to specific genes are first aggregated across all cells belonging to the same cell subpopulation (cell type) within each biological replicate [102]. Following this aggregation procedure, the resulting data structure and sequencing depth resemble bulk RNAseq profiles, enabling the application of well-established differential expression analysis tools such as edgeR, DESeq2, and limma-voom. According to several systematic benchmarking studies [103], pseudobulk methods demonstrate high stability, support standard statistical correction procedures, and provide strong statistical power for detecting subpopulation-specific gene expression changes. Data aggregation also reduces the impact of intra-population noise and sparsity characteristic of scRNAseq profiles, thereby increasing robustness to technical artifacts [103].

Thus, the pseudobulk approach is essential when comparing groups of scRNAseq samples, as it enables the aggregation of gene expression across cells within each sample and allows a proper estimation of inter-sample variability [4]. In addition, it has been demonstrated that incorporating scRNAseq-derived pseudobulk profiles into joint analyses with true bulk RNAseq datasets, followed by a projection of bulk data into the scRNAseq expression space (the SQUID method), substantially improves the accuracy of cellular composition deconvolution compared with routinely used tools [104]. Modern integrative Bayesian models, such as BayesPrism, extend this framework further by using scRNAseq data as prior information and simultaneously estimating both cell-type proportions and their internal expression profiles directly within each bulk RNAseq sample. By explicitly modeling technical and biological differences between bulk RNAseq and scRNAseq data, these approaches enhance deconvolution accuracy and enable a more reliable reconstruction of gene expression patterns in tissue samples [7].

Recent systematic benchmarking studies have shown that deconvolution methods should be compared not only through isolated applications, but across multiple independent datasets and experimental settings [105]. In such comparisons, performance is typically evaluated using quantitative metrics such as correlation with known cell-type proportions, root mean squared error, mean absolute error, robustness under mismatched-reference, or cross-platform conditions [106]. These studies also highlight substantial practical differences between methods, including runtime, memory usage, and input requirements [106,107]. Methods also differ substantially in their input requirements and modeling assumptions: for example, MuSiC was designed to use multi-subject scRNAseq references to account for cross-subject variability, CIBERSORTx relies on the construction of a signature matrix for the cell subsets of interest, and Bisque can further improve decomposition accuracy when matched bulk and single-cell or single-nucleus profiles are available to learn gene-specific transformations [6,54,95]. Bayesian methods such as BayesPrism use a probabilistic deconvolution framework, but this added modeling complexity can come at a substantial computational cost [108]. Collectively, these observations indicate that method selection should be guided not only by the underlying statistical framework, but also by benchmark performance, reference design, and practical feasibility in a given dataset.

### 3.3. Practical Applications of Integrative Analysis

Beyond their computational value, integrative bulk and single-cell transcriptomic approaches have important biological implications because they help disentangle several distinct sources of variation that are otherwise conflated in tissue-level expression profiles. In particular, they make it possible to distinguish whether an observed bulk transcriptomic change reflects altered cellular composition, transcriptional reprogramming within a specific cell population or a combination of both processes. This distinction is essential for interpretation of complex biological phenomena such as immune activation, stromal remodeling, differentiation, neurodegeneration, and therapy resistance, where shifts in cell abundance and changes in cell state often occur simultaneously. As a result, integrative analysis can reveal disease-relevant cellular programs, identify the likely cellular origin of bulk-derived biomarkers, and improve mechanistic interpretation of pathological processes that cannot be resolved from bulk or single-cell data alone.

Studies in melanoma and sarcomas have demonstrated that combining scRNAseq-derived signatures with deconvolution of bulk RNAseq data enables the reconstruction of the cellular composition of the tumor microenvironment and identification of clinically relevant cellular subtypes. In one example [94], scRNAseq signatures of T cells, B cells, macrophages, endothelial cells, and cancer-associated fibroblasts (CAFs) were used to deconvolve bulk RNAseq datasets. This approach allowed the characterization of melanoma cellular clusters and delineation of T-cell activation and exhaustion profiles, as well as CAF-associated tumor cell phenotypes. In another study [109], deconvolution of bulk RNAseq data enabled the identification of immune subtypes of sarcomas, among which a class characterized by high B-cell abundance and the presence of tertiary lymphoid structures (TLS) was associated with improved survival and increased sensitivity to PD-1 immune checkpoint blockade.

Beyond deconvolution of cellular composition, the integration of scRNAseq with bulk RNAseq can also improve interpretation of bulk-derived biomarkers and prognostic signatures by identifying their likely cellular origin. This is particularly important in heterogeneous tissues such as tumors, where bulk transcriptomic changes may reflect mixed epithelial, stromal and immune contributions rather than transcriptional shifts within a single cellular compartment. In such settings, scRNAseq can be used to prioritize cell-type-specific marker genes among bulk-dysregulated transcripts and thereby refine biologically interpretable prognostic models. For example, in prostate cancer, the integration of bulk transcriptomic cohorts with scRNAseq data was used to show that many consistently dysregulated genes were predominantly epithelial markers, which enabled the development of an epithelial cell-informed prognostic signature with improved risk stratification performance [24].

As demonstrated in recent immunogenomics studies, the integration of scRNAseq with bulk RNAseq enables the reconstruction of T-cell infiltration levels and identification of T-cell exclusion regions within tumors based on single-cell-derived signatures. When combined with large-scale analyses of bulk RNAseq datasets, such as the TCGA collection, this approach facilitates robust classification of immune tumor subtypes and the development of predictive models for survival and response to immune checkpoint inhibitor therapy [110,111]. In addition, integrated analysis of scRNAseq, bulk RNAseq, and ATAC-seq in head and neck squamous cell carcinomas demonstrated that early adaptive responses to cetuximab are heterogeneous and cell-type-dependent, revealing transcriptional and chromatin changes in pathways associated with therapeutic resistance that are not readily captured by bulk measurements alone [112]. In colorectal cancer, the integration of large-scale single-cell and bulk transcriptomic data identified two intrinsic epithelial tumor states, termed iCMS2 and iCMS3, and demonstrated that these states refine bulk-based consensus molecular classification and prognostic stratification, with CMS4 tumors harboring iCMS3 epithelium showing the poorest relapse-free survival [113].

Conversely, integrative transcriptomic approaches address several important challenges in neurobiology. For example, they enable attribution of observed gene expression changes to specific brain cell types (such as microglia, astrocytes, oligodendrocytes, and distinct neuronal subtypes), which is critical for understanding cellular mechanisms of neurodegeneration. Application of single-nucleus RNA sequencing (a specific implementation of scRNAseq) to the prefrontal cortex of patients with Alzheimer’s disease identified a disease-associated microglial state (Mic1) and neuronal subtypes exhibiting pronounced transcriptional vulnerability to amyloid and tau pathology. In bulk transcriptomic profiles, these cellular states were considerably less distinguishable due to the mixed contributions of multiple cell populations [114]. In a complementary large-scale analysis of Alzheimer’s disease, the integration of bulk brain RNAseq with single-nucleus transcriptomic references highlighted selective vulnerability of somatostatin interneurons and intra-telencephalic excitatory neurons and further linked these cell-type shifts to amyloid and tau burden, cognitive decline, and residual cognition [115].

In another study, the bioinformatic method Expression Weighted Cell-type Enrichment (EWCE) was shown to enable an interpretation of bulk gene expression and genetic data through the framework of reference single-cell transcriptomes, thereby identifying cell types that contribute most significantly to the pathogenesis of neurodegenerative diseases [116]. Similar strategies are applied in contemporary studies of psychiatric disorders [117], where scRNAseq data are used to define and spatially map cell-type-specific expression profiles underlying disease pathophysiology that may be obscured in bulk RNAseq datasets.

In cardiology, scRNAseq analysis of cardiac interstitial cells following myocardial infarction enabled a detailed characterization of cellular mechanisms underlying remodeling and inflammation, tracing the dynamic states of epicardial and endocardial fibroblasts and myofibroblasts, as well as immune and endothelial populations involved in scar formation and determining the risk of ventricular wall rupture across different mouse genetic backgrounds [118]. In transplantation research, single-cell RNAseq of kidney allograft biopsies has been used to derive cell-type-specific signatures for the deconvolution of independent bulk transcriptomic datasets, linking *FCGR3A*+ monocytes and *FCGR3A*+ NK cells to the severity of intragraft inflammation and antibody-mediated rejection [119].

Taken together, these examples illustrate that the biological value of integrative transcriptomics lies not only in improved cell-type quantification, but also in its ability to link tissue-level molecular phenotypes with specific cellular states, interactions, and disease mechanisms.

Thus, the integration of bulk RNAseq and scRNAseq data is becoming a standard approach in biomedical research, enabling the linkage of tissue cellular architecture with population-scale and clinical transcriptomic datasets and enhancing interpretability of the results. The growing number of deconvolution methods, continued refinement of pseudobulk gene expression models, and emergence of novel Bayesian algorithms position this field as one of the major directions in the development of modern experimental biomedicine.

## 4. Bioinformatic Tools for Integration

The development of integrative transcriptomic approaches has been driven by advances in computational tools, enabling the processing of scRNAseq data, generation of reference cell-type signatures, and deconvolution of bulk RNAseq datasets. In contrast to classical transcriptomic analysis, where the primary objective is identification of differentially expressed genes, the integration of multi-level RNA sequencing data requires a hierarchical analytical framework. This includes the accurate characterization of the structural properties of scRNAseq data space and effective transfer of this information to aggregated yet statistically robust bulk RNAseq datasets. In this section, we review the algorithmic principles underlying modern integration methods, including deconvolution approaches, scRNAseq analysis tools, and commonly used computational frameworks. Particular attention should be paid to the practical limitations, hidden assumptions, potential sources of bias that affect the performance, and interpretation of these methods in real-world datasets.

### 4.1. Deconvolution Methods

The MuSiC (MUlti-Subject Single-Cell) deconvolution method [95] was proposed as a deconvolution approach that uses scRNAseq data obtained from multiple donors as reference, thereby accounting for inter-individual variability in gene expression across analyzed cell types. In addition, MuSiC models stochastic intercellular variability within each cell type, enabling more accurate transfer of cell-type-specific expression profiles across datasets and reducing the influence of marker genes shared among multiple cell types. A key feature of MuSiC is its hierarchical deconvolution strategy: it first estimates the abundance of major related cell clusters and subsequently resolves individual subtypes within each cluster, thereby improving accuracy when analyzing closely related cell populations.

The SCDC method [120] extends the MuSiC deconvolution framework by incorporating multiple independent reference datasets into the analysis. Deconvolution is performed separately for each reference and the resulting estimates are subsequently combined using optimized weighting schemes. According to the authors, this strategy more effectively accounts for inter-dataset variability and provides improved accuracy compared with the standard implementation of MuSiC.

CIBERSORTx [6] represents an extension of the original CIBERSORT method, which is based on ν-support vector regression and incorporates additional cross-platform normalization modules to mitigate platform-specific differences between reference profiles and bulk RNAseq transcriptomes. A key feature of CIBERSORTx is its ability to reconstruct cell-type-specific expression profiles from bulk RNAseq datasets (in silico purification). The authors demonstrated this capability in cohorts of follicular lymphoma, diffuse large B-cell lymphoma, non-small-cell lung cancer, and melanoma. The reconstructed profiles showed strong concordance with expression profiles obtained from sorted cell populations using flow cytometry and enabled the identification of distinct functional cell states, including those associated with specific mutations. As an example of practical application, Qi et al. [121] used scRNAseq-derived reference profiles together with CIBERSORTx and MuSiC to deconvolve bulk RNAseq data from more than 500 head and neck squamous cell carcinomas and showed that higher T-cell infiltration, particularly a stronger regulatory T-cell signal, was associated with improved overall survival. Meanwhile, the classical CIBERSORT method continues to be widely applied in contemporary studies of the tumor microenvironment; for example, in a recent study [122] it was used to assess immune infiltration and to identify differences in T-cell, NK-cell, macrophage, and neutrophil populations between patient groups with high and low prognostic risks for colorectal cancer.

The Bisque method [54] is designed for deconvolution using scRNAseq reference data in scenarios where substantial platform-specific discrepancies exist between bulk RNAseq and scRNAseq measurements for certain genes. As a key step, Bisque constructs a reference profile from scRNAseq data, generates pseudobulk expression profiles, and estimates gene-specific linear transformations that align expression levels in bulk RNAseq profiles with the corresponding pseudobulk data. Deconvolution is then performed on the corrected bulk RNAseq profiles. In a recent systematic evaluation of multiple deconvolution methods across eight datasets containing matched bulk RNAseq and scRNAseq profiles, approaches incorporating the linear transformation of bulk profiles into the scRNAseq expression space, following the strategy proposed in Bisque and combined with a weighted least squares framework (SQUID), demonstrated higher accuracy in estimating cellular composition compared with direct deconvolution without such adjustment [104].

The xCell method [123] should be noted separately as a gene signature-based approach that computes enrichment scores for 64 immune and stromal cell types based on sample-level expression profiles. The resulting enrichment scores are transformed onto a linear scale but do not represent absolute cell-type proportions; therefore, xCell is generally classified as an enrichment analysis method rather than a classical deconvolution algorithm. xCell incorporates a spillover compensation mechanism to reduce artificial correlation between closely related cell populations (e.g., CD4^+^ and CD8^+^ T cells, monocytes, and neutrophils), making it suitable for comparing relative cell-type representation across sample groups. For example, application of xCell to 9947 tumor profiles from the TCGA and Therapeutically Applicable Research To Generate Effective Treatments (TARGET) databases generated a composite microenvironment score that inversely correlated with tumor cellularity estimates and enabled a comparison of microenvironment composition across different tumor types [123].

The Kassandra method [96] represents one of the most advanced deconvolution algorithms, based on training an ensemble of decision tree-based models (LightGBM) on millions of artificially generated transcriptomes that simulate biological variability, technical noise, and aberrant tumor cell expression patterns. This design enables robust performance in scenarios with overlapping marker profiles and improves the discrimination of closely related immune subpopulations, including multiple T-cell, NK-cell, and macrophage subtypes. According to multi-level orthogonal experimental validation, Kassandra outperforms existing methods in terms of accuracy and robustness of tumor microenvironment reconstruction, including clinically relevant components, such as CD8^+^ T cells associated with response to immunotherapy. An earlier proposed approach [9] demonstrated the conceptual feasibility of constructing accurate digital “artificial transcriptomes” for training deconvolution algorithms, providing the methodological foundation for subsequent development of Kassandra.

BayesPrism [7] is a Bayesian deconvolution approach that uses scRNAseq data as a prior reference and jointly estimates cell-type proportions and their mean expression profiles within each bulk RNAseq sample. The probabilistic framework accounts for within-cell-type variability as well as systematic differences between scRNAseq and bulk RNAseq profiles, thereby increasing the robustness of deconvolution to cross-platform discrepancies. BayesPrism demonstrates high accuracy in reconstructing gene expression in both malignant and non-malignant cells and exhibits strong reproducibility. However, a notable limitation of the method is its dependence on the completeness of the reference scRNAseq dataset, as missing cell types or cellular states cannot be accurately inferred. Deep learning-based deconvolution represents an emerging strategy for estimating cell-type composition from bulk transcriptomic data [124].

Unlike classical regression-based frameworks, these methods learn nonlinear relationships between bulk expression profiles and cell-type proportions, typically using synthetic bulk mixtures generated from scRNAseq data for training. A representative example is Scaden, a deep neural network-based method trained on simulated mixtures derived from scRNAseq data [97]. However, recent large-scale benchmarking and methodological review indicate that such approaches should currently be viewed as complementary rather than universally superior alternatives, because their performance remains dependent on reference design, training data quality, dataset-specific biological, and technical factors [124].

Collectively, these methods differ not only in algorithmic framework, but also in the assumptions they make about reference quality, cross-platform comparability, and the degree to which cellular states can be represented as discrete classes. As a result, their performance may vary substantially depending on tissue complexity, the completeness of the reference atlas. From our perspective, no currently available deconvolution method can be regarded as universally optimal across all biological contexts. In practice, methods such as MuSiC may be particularly useful when multi-subject reference datasets are available and inter-individual variability is expected to be substantial, whereas Bisque may be preferable in the presence of pronounced cross-platform discrepancies between bulk and single-cell profiles. Bayesian approaches such as BayesPrism are especially attractive when the analytical goal extends beyond estimation of cell-type proportions to reconstruction of cell-type-specific expression programs.

More generally, the robustness of deconvolution results depends strongly on the compatibility between the scRNAseq reference and the target bulk RNAseq dataset, as well as on the presence of missing, rare, or transcriptionally overlapping cell populations in the reference atlas. Therefore, in practice, method selection should be guided not only by reported benchmark performance, but also by reference design, tissue complexity, cross-platform compatibility, and the specific analytical objective, such as the estimation of relative cell fractions versus inference of cell-type-specific expression programs. The key characteristics of these methods are summarized in Table 2.

#### Practical Considerations for Method Selection and Unresolved Challenges

In addition to their algorithmic differences, deconvolution methods should also be compared from a practical perspective, including their performance across independent datasets, computational burden, runtime, and input requirements. In practice, these features may substantially affect method selection, particularly when integrating bulk RNAseq with scRNAseq references that differ in annotation depth, donor composition, and technical platform.

Despite the availability of multiple deconvolution algorithms, no single method can be considered universally optimal across all biological settings. Their performance is influenced not only by differences in algorithmic design, but also by practical limitations and potential sources of bias, including incomplete references, platform mismatch, tissue complexity, and uncertainty in the statistical interpretation of inferred cell fractions. Accordingly, method selection depends on several interacting factors, including the design and completeness of the scRNAseq reference, the extent of technical mismatch between bulk RNAseq and scRNAseq data, and the complexity of the analyzed tissue [104]. In particular, an important distinction should be made between studies aimed primarily at estimating cell-type proportions and those aimed at reconstructing cell-type-specific expression profiles from bulk transcriptomic data [125].

To facilitate method selection, Table 3 summarizes the published quantitative benchmark performance, approximate computational runtime, and input format requirements for the deconvolution methods discussed above. Performance is reported as Pearson correlation coefficient (*r*) or concordance correlation coefficient (CCC) with ground-truth cell-type proportions, as available from original validation or independent benchmark studies. These values should be interpreted with caution, as benchmark outcomes are sensitive to dataset composition, reference design, and the availability of true ground-truth proportions. Notably, runtime estimates reflect the processing of approximately 5000 reference cells and may scale substantially with dataset size, particularly for computationally intensive methods.

Recent comparative benchmarking studies [129] further indicate that deconvolution performance depends not only on the algorithm itself but also on several practical design choices, including the strategy used to construct the single-cell reference, the degree of match between cell types present in the reference and in the bulk sample, the size of the reference dataset, and the level of cell-type subdivision. These factors can substantially alter both the accuracy and robustness of estimated cell fractions and therefore should be considered explicitly when selecting a deconvolution framework and interpreting its output.

As a practical guideline, MuSiC may be preferable when the available scRNAseq reference includes multiple subjects and inter-individual variability is expected to be substantial, as the method explicitly models for cross-subject heterogeneity in cell-type-specific gene expression [95]. By contrast, when bulk RNAseq and scRNAseq profiles are generated using different platforms or protocols and exhibit pronounced systematic discrepancies, methods that are more robust to assay-specific differences (such as Bisque) may be more appropriate [130]. If the analytical goal extends beyond estimating cellular proportions to the joint inference of cell-type fractions and cell-type-specific gene expression, methods such as BayesPrism may be particularly useful [7]. In highly heterogeneous tumor samples, machine learning-based approaches such as Kassandra may provide greater robustness, particularly when technical and biological variability and aberrant malignant gene expression complicate deconvolution [96]. Accordingly, xCell [123] is most appropriately used to identify relative shifts in immune and stromal enrichment across samples, whereas methods designed for explicit proportion estimation are preferable when absolute or directly comparable cell fractions are required.

Several unresolved methodological challenges remain common to nearly all current deconvolution frameworks. First, deconvolution accuracy depends critically on the completeness of the reference atlas and missing cell types or states in the reference may systematically bias inferred composition, particularly when the missing populations are transcriptionally similar to those retained in the reference [131]. Second, closely related cell subtypes and continuous cellular state transitions remain difficult to resolve, because fine-resolution deconvolution is still methodologically challenging and many cell populations, particularly in tumors, occupy continuous rather than discretely separable phenotypic manifolds [132,133]. Third, reference profiles often transferred across studies, donors, sequencing protocols, and deconvolution robustness may decline when cross-subject heterogeneity or assay-specific technical biases weaken the correspondence between the single-cell reference and the target bulk transcriptome [54,95]. The statistical interpretation of estimated cell fractions remains nontrivial because these quantities are compositional: their non-negativity and sum-to-one constraints induce mutual dependence between components, so an increase in one inferred fraction necessarily affects the relative values of the others [134]. These limitations indicate that deconvolution results should generally be interpreted as model-dependent estimates whose reliability depends on reference design, tissue biology, platform compatibility, and the assumptions of the selected method, rather than as algorithm-independent measurements of true cellular composition [122]. Independent benchmark studies [130] have also shown that performance rankings are not fully stable across tissues and experimental settings, in part because reliable ground-truth cell proportions are rarely available and because benchmarking outcomes are sensitive to reference dataset bias, marker-gene selection, tissue-specific heterogeneity, and differences in RNA extraction and library preparation strategies.

Beyond method selection, it is equally important to recognize scenarios in which bulk-scRNAseq integration should not be performed or should be interpreted with particular caution. First, when the scRNAseq reference and the bulk RNAseq dataset originate from different tissues or anatomical compartments, deconvolution estimates are likely to be biologically unreliable: as explicitly formulated in the design assumptions of recent deconvolution frameworks, reference and target data must be derived from the same tissue or organ to ensure that relevant cell types are represented [99]. Second, integration may be unreliable when reference and bulk data correspond to different disease states. Feng et al. note that deconvolving disease samples with healthy references requires careful consideration of disease-specific cell types that may be absent from the reference [100]; and controlled resilience analyses confirm that the performance of reference-based methods declines substantially when reference and bulk data originate from independent datasets with differing biological or technical contexts [135]. Third, when batch effects between the reference and the target dataset are severe and cannot be adequately corrected by available harmonization approaches, deconvolution outputs may primarily reflect technical rather than biological variation. Cross-reference benchmarking across multiple tissue types has demonstrated that this setting substantially reduces deconvolution accuracy compared with idealized self-reference settings, even for top-performing algorithms [100,135].

Fourth, when the scRNAseq reference atlas is substantially incomplete (omitting cell types present in the bulk sample), deconvolution will redistribute signal from the missing populations onto retained ones, systematically biasing all estimated fractions; this effect worsens with the number and proportion of missing cell types and their signal may remain detectable in deconvolution residuals [105,131]. Fifth, the compositional constraints inherent to deconvolution outputs mean that an increase in one inferred fraction necessarily affects all others [134], rendering results particularly unreliable in near-pure or very low-diversity samples. In such scenarios, deconvolution results should be interpreted as model-dependent estimates rather than direct measurements of cellular composition. Independent validation using orthogonal approaches such as immunohistochemistry, flow cytometry, or spatial transcriptomics is, therefore, recommended before drawing biological or clinical conclusions [136].

### 4.2. scRNAseq Reference Preparation Tools for Bulk–Single-Cell Integration

High-quality scRNAseq data analysis is a prerequisite for robust integration with bulk RNAseq datasets, because the accuracy of downstream deconvolution and related integrative approaches critically depends on the quality of cell clustering, annotation, batch correction, and reference signature construction. Although not all of the tools discussed below are direct bulk–single-cell integration methods, they play an essential role in generating reliable scRNAseq-derived reference atlases and harmonized cell-type profiles required for subsequent integrative analyses. An increasingly important source of reference information for bulk and single-cell integration is provided by large cell atlas projects. Such resources expand the scale, tissue coverage, and annotation depth of available reference data, enabling the construction of more comprehensive reference matrices and improving representation of diverse, including rare or tissue-specific, cell populations [137]. In practice, atlas-scale references can facilitate cross-study harmonization and serve as broad reference frameworks when a study-specific single-cell dataset is not available [138]. At the same time, their use introduces additional challenges, including differences in donor composition, tissue sampling, experimental protocols, annotation granularity, batch effects, and computational burden, which require careful harmonization and context-aware reference selection [139]. The Seurat [140] and Scanpy [141] packages represent widely used platforms for such analyses.

Scanpy provides scalable workflows for normalization, dimensionality reduction, graph-based clustering, and marker gene identification, whereas Seurat supports feature selection, graph-based cell-state representation, and multimodal atlas construction through weighted nearest neighbors. In this context, such frameworks are particularly relevant because they enable the generation of robust cell clusters and reference signatures for downstream deconvolution and related integrative analyses [141,142].

For the harmonization of scRNAseq datasets generated in independent experiments, methods such as Harmony [143], LIGER [144] and scVI [145] are frequently applied. Harmony performs batch correction in a precomputed low-dimensional embedding, thereby promoting clustering according to biological similarity rather than technical source. LIGER uses integrative non-negative matrix factorization to identify shared and dataset-specific expression factors, which is useful for constructing reference atlases across samples, tissues, or platforms. A representative application of this strategy was reported by Oh et al., who integrated 92 scRNAseq samples from seven independent studies of pancreatic ductal adenocarcinomas using a Seurat-based workflow with Harmony batch correction, enabling the construction of a reproducible cross-study tumor microenvironment atlas and identification of subtype-associated differences in fibroblast, macrophage, and T-cell populations [146]. ScVI applies a probabilistic latent-variable framework to model sparse UMI counts while accounting for batch effects and library size and is particularly attractive for large-scale reference construction. Together, these methods improve the consistency and cross-study comparability of scRNAseq-derived reference profiles used in bulk and single-cell integration.

Analysis using the SCENIC method [147] based on scRNAseq data enables the simultaneous reconstruction of gene regulatory networks and estimation of regulon activity (sets of genes coordinately regulated by the same transcription factor) in individual cells, facilitating the identification of key transcription factors and stable cellular states. The resulting regulon activity matrix provides a biologically interpretable dimensionality reduction framework, upon which the authors demonstrate improved cell clustering accuracy and reduced influence of technical artifacts, including clustering driven by sample origin or analytical platform rather than true cell-type identity. In the context of bulk and single-cell integration, regulon activity scores derived via SCENIC can serve as cell-type-specific transcriptional signatures, providing a mechanistic context for transcription factor-driven expression changes that deconvolution of bulk RNAseq data attributes to particular cell populations.

Automated annotation tools are also relevant for reference construction and label transfer across datasets. For example, SingleR [148] annotates individual cells by comparing their transcriptomes with reference expression profiles and can support the standardized assignment of cell identities in scRNAseq-derived atlases used for downstream deconvolution. CellTypist [149] is another useful annotation framework, particularly for standardized immune cell labeling across large scRNAseq datasets. At the preprocessing stage, quality-control procedures may also include the detection of technical doublets, for example with tools such as Scrublet [150], to reduce the risk of artifactual mixed-cell profiles in the reference dataset.

Collectively, the tools described above constitute a modern toolkit for scRNAseq preprocessing, annotation, harmonization, and downstream biological interpretation (Table 4). Although not all of these methods are direct bulk RNAseq and scRNAseq integration tools, they are highly relevant for integrative transcriptomics because they support the construction of high-quality reference atlases and improve the robustness of subsequent deconvolution and related analytical workflows.

### 4.3. Example of an Analytical Pipeline for Integrating Bulk RNAseq and scRNAseq Data

**Figure 4:**
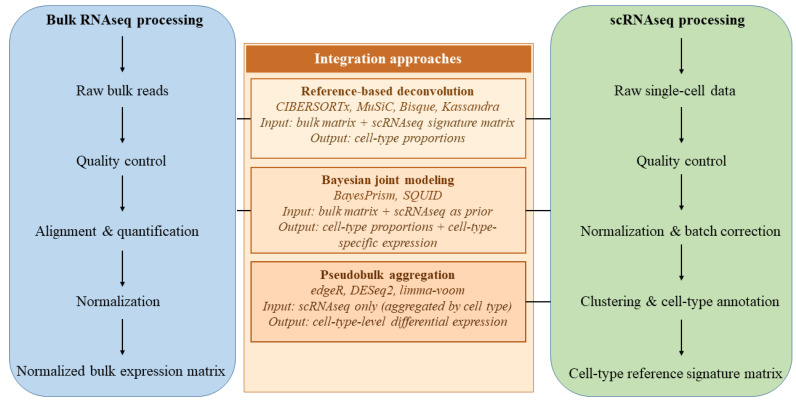
Example computational workflow for integrative analysis of bulk and scRNAseq data. Bulk RNAseq data undergo standard preprocessing, including quality control (QC), read alignment, and quantification and normalization, resulting in a normalized bulk expression matrix. In parallel, scRNAseq data are processed through quality control, normalization, batch correction, and clustering, followed by cell-type annotation and construction of a cell-type reference signature matrix. These outputs serve as inputs for three principal integration approaches, which differ in their input requirements and analytical outputs: (1) reference-based deconvolution (CIBERSORTx, MuSiC, Bisque, Kassandra), which requires both a bulk expression matrix and a scRNAseq-derived signature matrix and estimates cell-type proportions; (2) Bayesian joint modeling (BayesPrism, SQUID), which uses scRNAseq data as a probabilistic prior together with the bulk matrix and jointly estimates cell-type proportions and cell-type-specific expression profiles; and (3) pseudobulk aggregation (edgeR, DESeq2, limma-voom), which operates on scRNAseq data aggregated by cell type and enables cell-type-level differential expression analysis without requiring bulk RNAseq input.

Integrative analysis is typically performed as a sequential computational workflow aimed at translating bulk (aggregated) transcriptomic profiles into tissue models with cellular resolution. The first stage involves quality control, including the removal of cells with an excessively low number of detected genes or flagged as low quality based on other technical metrics. This is followed by the normalization of gene expression count matrices (e.g., scaling library size to 10,000 UMI or reads per cell, log transformation, and gene-wise z-score scaling). Subsequently, integration methods are applied to mitigate batch effects and generate a harmonized representation of the data, such as anchor-based integration implemented in Seurat v3 or low-dimensional embedding correction using Harmony [143,151]. An example of a practical computational workflow for integrating bulk RNAseq and scRNAseq data is illustrated in Figure 4.

Bulk RNAseq data undergo their own quality control procedures, including technical filtering, read alignment, and normalization. For example, in a study [152], the analysis of bulk RNAseq profiles began with a gene-level read count matrix generated after alignment using TopHat2 and quantification of uniquely mapped reads. Subsequently, DESeq2 was applied to normalize gene-level read counts, followed by regularized log transformation (rlog) and clustering to assess sample quality and underlying data structure.

High-quality scRNAseq data are used to construct structured representations of cellular populations: high-dimensional transcriptional profiles enable the identification of cell clusters, while UMAP provides a compact and interpretable visualization for downstream analysis [153]. The accuracy and robustness of the reference directly determine the performance of subsequent deconvolution steps, as demonstrated in a comparative study [105], which showed that the omission of even a single cell type from the reference matrix leads to a marked decline in estimation accuracy, regardless of the selected deconvolution method or prior data transformation procedures.

Following the construction of reference signatures, the deconvolution of bulk RNAseq data is performed as the key step of the integrative analysis. The specific methods and their algorithmic features have been discussed above; here it is important to emphasize that, regardless of the chosen approach, whether classical regression-based models, signature-based methods, or modern machine learning algorithms, the fundamental objective of deconvolution remains the same: to reconstruct tissue cellular composition and, when applicable, infer cell-type-specific expression profiles. As highlighted in methodological studies on transcriptomic deconvolution (e.g., [97]), researchers often apply multiple deconvolution algorithms in parallel and compare their outputs on identical datasets to assess robustness of conclusions across methodological frameworks. The resulting estimates of cellular composition then form the basis for biological interpretation, enabling the explicit consideration of cell-type contributions to the bulk transcriptome and improving the accuracy of downstream analyses [129]. For example, Zhang et al. [154] applied an integrative workflow in colorectal cancer combining scRNAseq preprocessing and clustering, Harmony-based batch correction, SCENIC regulon analysis, Monocle2 trajectory inference, CellChat-mediated intercellular communication analysis, and CIBERSORT-based deconvolution of TCGA bulk RNAseq data, which enabled the identification of immune cell subpopulations, definition of five tumor microenvironment subtypes, and evaluation of their prognostic significance.

Subsequent integration of transcriptomic data enables the identification of the cellular composition of a tissue and supports interpretation of transcriptomic alterations in the context of underlying cell-type contributions. Thus, a typical analytical pipeline for integrating bulk RNAseq and scRNAseq data represents a multi-stage workflow encompassing data preprocessing, construction of cell-type signatures, deconvolution, functional interpretation, and, ideally, subsequent experimental or independent analytical validation.

## 5. Conclusions

In this review, we described the principal strategies for integrating different types of transcriptomic data, namely aggregated bulk RNAseq profiles and single-cell transcriptomes generated by scRNAseq. We outlined the strengths and limitations of each individual approach and demonstrated how their integration can qualitatively and quantitatively enhance transcriptomic analysis. Importantly, the value of bulk and single-cell integration extends beyond an estimation of cell-type proportions alone. In many biomedical settings, these approaches also provide a conceptual bridge between cell-resolved biology and clinically scalable bulk transcriptomics by improving the interpretation of biomarker signals, reference-guided analysis, and the biological attribution of transcriptomic changes.

In summary, the central conceptual contribution of this review is the systematic treatment of bulk and single-cell integration as a distinct analytical discipline rather than a simple extension of either technology alone. We tried to provide here a structured comparative framework for integration methods evaluated across their algorithmic assumptions, quantitative performance, computational requirements, and practical applicability. We identify three key conceptual insights that emerge from this synthesis: (1) no single deconvolution method is universally optimal and method selection must be guided by reference design, cross-platform compatibility, and the specific analytical objective; (2) the accuracy of any integration approach is fundamentally constrained by the completeness and representativeness of the scRNAseq reference atlas, making reference construction a critical (yet often underappreciate) determinant of analytical success; and (3) integrative analysis provides biological value beyond cell-type quantification alone, enabling the attribution of bulk-derived biomarker signals to specific cellular origins and distinguishing compositional shifts from transcriptional reprogramming within individual cell populations.

Future progress in this field will likely depend on the development of more context-specific reference atlases, improved modeling of continuous and transitional cell states, broader integration with multimodal and spatial single-cell data, and more realistic benchmarking under cross-platform and incomplete-reference conditions. Under these conditions, bulk–single-cell integration is expected to further improve the mechanistic interpretation of tissue transcriptomes, biomarker discovery, and translational applications.

## Figures and Tables

**Table 1:** Comparative characteristics of scRNAseq and bulk RNAseq.

Parameter	Bulk RNAseq	scRNAseq
Level of resolution	Tissue/cell population	Single cell
Sequencing depth	10–30 million reads per sample [33]	10,000–100,000 reads per cell [58,68]
Dynamic range of detection	Wide dynamic range; enables detection of low- and high-abundance transcripts [69]	Lower effective dynamic range at the single-cell level; limited by low mRNA capture efficiency and dropout events; no broadly accepted quantitative estimate [62,63]
Proportion of zero values in count matrix	Moderate (~10–40%) [70,71]	High (~80%) [64]
Resolution of cellular heterogeneity	Not resolved (signal averaging) [41,44]	Fully resolved [3,60]
Detection of rare cell populations	Limited [6]	Feasible given sufficient cell numbers [3,60]
Inter-laboratory reproducibility	High (correlation >0.9) [72]	Variable depending on protocol [73]
Sample requirements	Fresh, frozen or formalin-fixed paraffin-embedded (FFPE) tissue [74]	Primarily fresh or cryopreserved samples [13,14]
Relative cost	Moderate [75]	High [75]
Scalability for cohort studies	High (thousands of samples) [38,39,40,76,77]	Limited [75]
Major technical artifacts	Minimal	Amplification bias [62], dissociation-induced stress [65], loss of sensitive populations [13,78]

**Table 2:** Key characteristics of selected deconvolution methods.

Method	Year	Algorithmic Approach	Main Advantages	Limitations
CIBERSORTx [6]	2019	ν-support vector regression (ν-SVR) with cross-platform normalization	Enables construction of custom signatures using scRNAseq data; reconstructs cell-type-specific expression profiles	High computational requirements; requires a predefined signature matrix
MuSiC [95]	2019	Weighted non-negative regression accounting for inter-individual and intra-cell-type variability	Accounts for sample-level and within-cell-type variability, improving accuracy, especially for closely related cell types	Requires scRNAseq reference profiles from multiple donors
SCDC [120]	2021	Integration of multiple scRNAseq reference datasets with optimized weighting	Improves accuracy through ensemble use of multiple reference datasets	Performance depends on consistency between reference datasets
Bisque [54]	2020	Gene-specific linear transformation with weighted least squares adjustment	Corrects systematic discrepancies between bulk RNAseq and scRNAseq data, improving gene-level accuracy	Optimal performance when paired bulk RNAseq and scRNAseq data are available
BayesPrism [7]	2022	Bayesian probabilistic model	Jointly estimates cell proportions and cell-type-specific expression profiles; accounts for within-cell-type variability and cross-platform differences	Computationally intensive; sensitive to incompleteness of the reference scRNAseq dataset
Kassandra [96]	2022	Gradient boosting using LightGBM trained on simulated transcriptomes	High accuracy and robustness; performs well with overlapping markers and complex tissues (e.g., tumors)	Performance depends on the predefined training cell-type panel
xCell [123]	2017	Marker gene signature-based enrichment scoring	Reduces artificial correlation between related cell types; does not require external reference datasets; provides relative enrichment estimates	Does not provide absolute cell proportions; limited to enrichment-based interpretation
Scaden [97]	2020	Deep neural network trained on synthetic bulk mixtures derived from scRNAseq	Captures nonlinear relationships; competitive performance across simulated and experimental datasets; robust to noise and technical bias in some settings	Reduced interpretability; depends on training data design and transferability across datasets

**Table 3:** Quantitative benchmark performance, computational runtime, and input requirements of selected deconvolution methods.

Method	Typical Pearson *r* Range (Published Benchmarks)	Approx. Runtime (5000 Ref. Cells)	Bulk Input Format	scRNAseq Reference Requirement
CIBERSORTx [15]	*r* 0.69–0.97 on simulated mixtures across dynamic conditions, performance declines with increasing tumor purity [126]	~5 min [127]; Docker/web server required	Tab-delimited normalized expression matrix; Docker or web server	Predefined or custom scRNAseq signature matrix; cross-platform normalization built in
MuSiC [95]	Top-ranked on simulated and pseudobulk datasets [95,128]; performance variable on real bulk data (Pearson *r* < 0.4 in some real PBMC benchmarks [129])	<30 s; fastest combined runtime [127]; no separate signature build step	R ExpressionSet (counts or CPM)	Multi-donor scRNAseq ExpressionSet; multi-subject reference strongly recommended
Bisque [54,130]	*r* = 0.92 on matched datasets [54]; cor = 0.48–0.68 on real brain tissue across RNAseq protocols [130]; drops substantially under strong cross-platform mismatch	<30 s [127]; no separate signature build step	R ExpressionSet	scRNAseq ExpressionSet; matched bulk+scRNAseq improves gene-specific transformation accuracy; ≥4 donors in reference recommended for stable performance
BayesPrism [7]	Top-ranked in heterogeneous simulation settings and for granular immune lineages in tumors [129]; correlation with ground truth >0.95 for malignant cell gene expression at >50% tumor purity	~5 min (external benchmark [127]); scales substantially with dataset size; computationally intensive	R raw count matrix with cell-type labels	scRNAseq raw count matrix; sensitive to reference incompleteness
SCDC [120]	Pearson *r* = 0.99 on controlled cell-line mixtures [120]; improves MuSiC estimates when multiple independent references integrated via ENSEMBLE weighting	~120 s for 5000 cells [127]	R ExpressionSet	Single or multiple scRNAseq ExpressionSets; ENSEMBLE framework requires ≥2 independent references
Scaden [97]	CCC = 0.88–0.98 on simulated data (average CCC = 0.88 on PBMC, CCC = 0.98 on pancreas); CCC = 0.56–0.92 on real bulk datasets (PBMC and brain) [97]	~27 min total (training + data generation) [127]; prediction ~8 s; GPU reduces training ~3×	Python/CLI; count data in AnnData (.h5ad) format	scRNAseq data of the same target tissue required for simulation of training mixtures; tissue-specific model must be trained before each new application; no pre-trained universal model available
Kassandra [96]	*r* = 0.83–0.97 across original validation studies; superior accuracy vs. CIBERSORTx and Scaden in TME validation [96]	Not reported in independent benchmark studies	TPM-normalized bulk expression matrix; custom transcript filtering required	Pre-trained LightGBM ensemble; no user-supplied reference required; fixed cell-type panel
xCell [123]	Produces enrichment scores only, not comparable to proportion-based *r* metrics	Seconds; marker-based scoring, no regression step	FPKM/TPM-normalized bulk expression matrix; R package or web server	Not required; curated marker gene signatures for 64 immune and stromal cell types

Performance is reported as Pearson correlation coefficient (*r*) or concordance correlation coefficient (CCC) with ground-truth cell-type proportions derived from original validation or independent benchmark studies. Runtime estimates correspond to approximately 5000 reference cells; values may scale substantially with dataset size. xCell produces enrichment scores rather than absolute cell-type proportions and is therefore not directly comparable to proportion-based methods. Abbreviations: CCC, concordance correlation coefficient; TME, tumor microenvironment; PBMCs, peripheral blood mononuclear cells.

**Table 4:** Computational tools for scRNAseq preprocessing, harmonization, and annotation relevant to bulk and single-cell integration.

Tool	Algorithmic Framework	Main Relevance to Integrative Workflows	Key Considerations
Seurat [151]	PCA; k-nearest neighbor (kNN) graph; shared nearest neighbor (SNN) graph; graph-based clustering; anchor-based dataset integration; weighted nearest neighbors (WNN) for multimodal analysis	Preprocessing, clustering, batch correction, multimodal atlas construction, reference signature generation	Performance depends on parameter selection; memory-intensive for large datasets
Scanpy [141]	PCA; neighborhood graph construction; Louvain/Leiden clustering; scalable sparse matrix implementation	Large-scale preprocessing, dimensionality reduction, marker gene identification	Requires careful parameter tuning; multimodal support relies on additional modules
Harmony [143]	Iterative batch correction in low-dimensional embedding space	Removal of batch effects prior to joint dataset analysis	Risk of overcorrection if biological and batch effects are confounded
LIGER [144]	Integrative non-negative matrix factorization (iNMF)	Joint analysis of multiple datasets with separation of shared and dataset-specific factors	Requires optimization of factor number and regularization parameters
scVI [145]	Hierarchical Bayesian variational autoencoder; negative binomial modeling of UMI counts	Latent representation learning; batch correction; scalable integration of large scRNAseq datasets	Computationally demanding for very large datasets
SCENIC [147]	Gene regulatory network inference (*GENIE3*/*GRNBoost*) combined with regulon activity scoring (*AUCell*)	Biological interpretation of regulatory programs in annotated cell populations	Dependent on completeness of transcription factor annotations
SingleR [148]	Correlation-based iterative annotation using reference transcriptomic datasets	Automated cell-type annotation and reference mapping	Annotation accuracy depends on reference dataset quality
CellTypist [149]	Logistic regression classifier trained on immune reference atlas	Automated immune cell subtype annotation	Primarily optimized for immune cell populations
Scrublet [150]	Simulation-based doublet detection using kNN-based scoring	Identification and removal of technical doublets prior to downstream analysis	Reduced sensitivity in highly homogeneous datasets

## Data Availability

No new data were created or analyzed in this study. Data sharing is not applicable to this article.
